# Pericardial Relapse of Acute Lymphoblastic Leukemia (ALL)

**DOI:** 10.1155/2021/9953230

**Published:** 2021-11-25

**Authors:** Diana V. Maslov, Ambuga Badari

**Affiliations:** ^1^Ochsner Clinic Foundation, Department of Internal Medicine, 1514 Jefferson Highway, New Orleans, LA 70121, USA; ^2^The Gayle and Tom Benson Cancer Center, 1516 Jefferson Highway, Jefferson LA 70121, USA

## Abstract

Acute lymphoblastic leukemia (ALL) is a neoplasm of the B cell or T cell. Diagnosis is made by peripheral blood smear and bone marrow biopsy. Those with relapse/measurable residual disease (MRD) present with fever, weakness, fatigue, and easy bruising due to bone marrow infiltration (Kantarjian et al., 2017). A 59-year-old male with history of relapsed acute lymphoblastic leukemia and allogeneic stem cell transplant presented to the Emergency Department (ED) multiple times with shortness of breath. 2D Echo revealed recurrent pericardial effusion. His MRD was discovered in the pericardium. He underwent the creation of a pericardial window with cytology and culture which confirmed B cell lymphoblastic leukemia/lymphoma, consistent with relapsed disease. We present a case of a patient with B-ALL and MRD who presented with symptoms of shortness of breath. His MRD was discovered not in the bone marrow, but in the pericardium.

## 1. Introduction

Acute lymphoblastic leukemia (ALL) is a neoplasm of the B cell or T cell. Approximately two-thirds of all ALL are B cell phenotype (B-ALL). This is often a disease of children, with most cases occurring in those <6 years old and then there is a second peak in those >60 [1, 2]. Patients tend to have symptoms of fever, fatigue, infection, or easy/spontaneous bruising or bleeding. This is associated with anemia, neutropenia, and thrombocytopenia due to bone marrow involvement. Patients can also have hepatomegaly, splenomegaly, and lymphadenopathy [3]. Diagnosis is made by peripheral blood smear and bone marrow biopsy [3]. Treatment of initial ALL consists of chemotherapy; the drug and duration differ based on the mutation and risk of disease. Patients may undergo stem cell transplantation if they have refractory or advanced relapsed ALL [4–8].

Those with relapse or measurable residual disease (MRD) also most often present with symptoms of fever, weakness, fatigue, and easy bruising associated with bone marrow infiltration [9]. We present a case of a patient with B-ALL and MRD who presented with symptoms of shortness of breath. His MRD was discovered not in the bone marrow, but in the pericardium.

## 2. Case Presentation

A 59-year-old male with history of relapsed acute lymphoblastic leukemia and allogeneic stem cell transplant about one year prior presented to the Emergency Department (ED) with shortness of breath. He reported that his shortness of breath had been noted gradually over the previous 4 weeks and was worse with exertion.

Review of systems was positive for generalized weakness, cough, tonsillar exudate, and fever. Physical exam had no abnormal findings. In the ED, he had a platelet of 25 k (his baseline; normal range 150-350 k), CRP 22.8 (normal range), BNP 436 (normal range), LDH 568 (normal range), troponin 0.581 (normal range), and was Coronavirus 19 negative.

**Figure 1:**
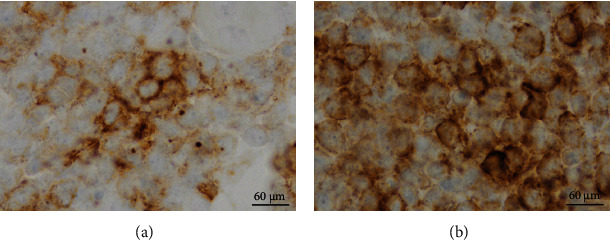
(a) Pericardial fluid CD10+. (b) Pericardial fluid CD19+.

**Figure 2:**
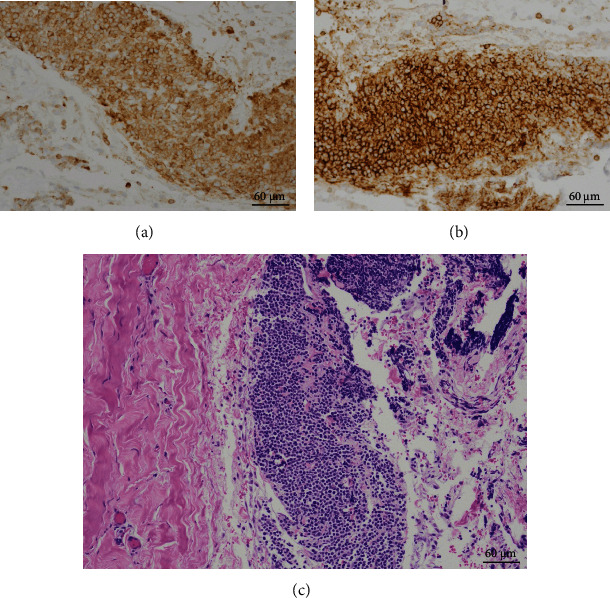
(a) Pericardial biopsy CD10+. (b) Pericardial biopsy CD19+. (c) Pericardial biopsy H&E stain.

Troponin continued to rise and 2D Echo revealed moderate pericardial effusion without tamponade. He was observed overnight and then discharged home with close hematology follow-up. He returned to the ED about 3 weeks later as another 2D Echo indicated worsening pericardial effusion. He had worsening shortness of breath over the previous five days with peripheral edema, weight gain, and abdominal distention. Labs showed anemia (hemoglobin 11.7) and thrombocytopenia (platelets 25). Review of systems was positive for epistaxis and bright red blood per rectum. Physical exam was positive for wheezes and 4+ pitting edema on bilateral lower extremities. He was admitted for management of pericardial effusion, and cardiothoracic surgery was consulted. He received 2 units of irradiated platelets. Repeat Echo showed large-sized pericardial effusion (1.1 cm circumferential effusion). He underwent the creation of a pericardial window the following day with cytology and culture. His symptoms were much improved and platelets (69) improved with transfusion and rituximab. Cytology showed B cell lymphoblastic leukemia/lymphoma, positive for CD19, CD10, CD5, CD13, TdT, and CD34, with 97.1% blasts, similar to the patient's original immunophenotype (Figure 1). This was consistent with relapsed disease. He underwent a bone marrow biopsy which was morphologically negative for ALL. Flow cytometry was unremarkable. MRD testing was positive for measurable residual disease. Pericardial biopsy confirmed gross disease (Figure 2). He was started on blinatumomab as salvage therapy.

## 3. Discussion

Acute lymphoblastic leukemia (ALL) is a neoplasm of the B cell or T cell that commonly affects those <6 years old or >60 [1, 2]. Approximately two-thirds of all ALL are B cell phenotype (B-ALL). The cause of B-ALL is unknown. B-ALL typically presents with “B-like symptoms” such as fatigue, night sweats, fevers, chills, weakness, and fatigue. This is due to bone marrow infiltration [3]. Diagnosis is made by looking at immunophenotype either in peripheral blood, bone marrow, or involved tissue. In B-ALL, lymphoblasts are almost always positive for CD19, CD79a, and CD22 [3]. Treatment of initial ALL consists of chemotherapy, although chemotherapy-free approaches are now being evaluated. Allogeneic stem cell transplantation is indicated for refractory or advanced relapsed ALL [4–8]. Upon initial diagnosis and treatment, our patient presented very similarly to the “standard” patient with ALL. His bone marrow was initially filled with lymphoblasts.

However, his relapse, just a year after his transplant, had a very unique presentation. Those with relapse or measurable residual disease (MRD) generally have similar symptoms of their initial presentation: fever, weakness, fatigue, and easy bruising [9]. About 5% of all ALL relapses occur in extramedullary (EM) sites. Those extramedullary relapses (EMRs) tend to involve the brain or testes [10]. Research has shown no significant difference in survival between those with bone marrow relapse vs. extramedullary relapse without bone marrow involvement [11]. Our patient presented with shortness of breath due to a pericardial effusion, and ultimately, he was diagnosed relapsed ALL involving the pericardium. There have been cases of unusual locations of relapse such as pelvic and gastric regions [12], but there has not been a reported case of ALL of the pericardium.

Interestingly, his bone marrow was morphologically normal, and only MRD was positive.

Blinatumomab is a monoclonal antibody also used in relapse and measurable residual disease (MRD). It is directed at CD19 on tumor cells. A phase 3 trial showed that it was superior to chemotherapy in regard to overall survival (OS), event-free survival (EFS), and complete remission (CR) rate [13]. The prognosis in adults with relapsed or refractory ALL is poor. Median survival is less than one year [14]. Our patient was treated with blinatumomab and achieved partial response, but subsequently progressed and opted for comfort care.

## Data Availability

Readers can access this data through PubMed or other study databases. All resources are in the References section.
